# Clinical characteristics, risk factors and outcomes of mixed *Candida albicans*/bacterial bloodstream infections

**DOI:** 10.1186/s12879-020-05536-z

**Published:** 2020-11-06

**Authors:** Li Zhong, Shufang Zhang, Kankai Tang, Feifei Zhou, Cheng Zheng, Kai Zhang, Jiachang Cai, Hongwei Zhou, Yesong Wang, Baoping Tian, Zhaocai Zhang, Wei Cui, Zhaohui Dong, Gensheng Zhang

**Affiliations:** 1 Department of Critical Care Medicine, Second Affiliated Hospital, Zhejiang University School of Medicine, Hangzhou, 310009 Zhejiang China; 2 Department of Critical Care Medicine, First Affiliated Hospital, Huzhou Teachers College, the First People’s Hospital of Huzhou, Huzhou, 313000 Zhejiang China; 3 Department of Cardiology, Second Affiliated Hospital, Zhejiang University School of Medicine, Cardiovascular Key Laboratory of Zhejiang Province, Hangzhou, 310009 Zhejiang China; 4 Department of Critical Care Medicine, Ningbo Medical Center, Li Huili Hospital, Ningbo, 315040 Zhejiang China; 5 Department of Critical Care Medicine, Taizhou Municipal Hospital, Taizhou, 318000 Zhejiang China; 6 Clinical Microbiology Laboratory, Second Affiliated Hospital, Zhejiang University School of Medicine, Hangzhou, 310009 China

**Keywords:** Candidemia, Bloodstream infections, Mixed Candida/bacterial bloodstream infections, *Candida albicans*, Mortality, Risk factor

## Abstract

**Purpose:**

The purpose of this study was to explore the clinical features, risk factors, and outcomes of mixed *Candida albicans*/bacterial bloodstream infections (mixed-CA/B-BSIs) compared with monomicrobial *Candida albicans* bloodstream infection (mono-CA-BSI) in adult patients in China.

**Methods:**

All hospitalized adults with *Candida albicans* bloodstream infection (CA-BSI) were recruited for this retrospective observational study from January 1, 2013, to December 31, 2018.

**Results:**

Of the 117 patients with CA-BSI, 24 patients (20.5%) had mixed-CA/B-BSIs. The most common copathogens were coagulase-negative *Staphylococcus* (*CNS*) (24.0%), followed by *Klebsiella pneumoniae* (20.0%) and *Staphylococcus aureus* (16.0%). In the multivariable analysis, a prior ICU stay > 2 days (adjusted odds ratio [OR], 7.445; 95% confidence interval [CI], 1.152–48.132) was an independent risk factor for mixed-CA/B-BSIs. Compared with patients with mono-CA-BSI, patients with mixed-CA/B-BSIs had a prolonged length of mechanical ventilation [17.5 (4.5, 34.8) vs. 3.0 (0.0, 24.5), *p* = 0.019] and prolonged length of ICU stay [22.0 (14.3, 42.2) vs. 8.0 (0.0, 31.5), *p* = 0.010]; however, mortality was not significantly different.

**Conclusions:**

There was a high rate of mixed-CA/B-BSIs cases among CA-BSI cases, and *CNS* was the predominant coexisting species. A prior ICU stay > 2 days was an independent risk factor for mixed -CA/B-BSIs. Although there was no difference in mortality, the outcomes of patients with mixed -CA/B-BSIs, including prolonged length of mechanical ventilation and prolonged length of ICU stay, were worse than those with mono-CA-BSI; this deserves further attention from clinicians.

## Introduction

In critically ill patients, bloodstream infection (BSI) is an important cause of morbidity and mortality. Candidemia is a leading cause of healthcare-associated BSI, with all-cause in-hospital mortality reaching 30% in the United States [1–3]. With the extensive use of antibiotics and immunosuppressants and rapid increases in the applications of invasive medical examinations and treatments, *Candida* has gradually become a significant pathogen responsible for BSI, with a crude average incidence of 8.7 per 100,000 population [2]. Patients with candidemia have many typical risk factors, including recent surgery, use of broad-spectrum antibiotics, presence of a central venous catheter (CVC), and injection drug use [2].

Although many candidemia cases are monomicrobial, mixed *Candida*/bacterial BSIs account for 18–56%, as previously reported [4–8]. In these studies [4–6, 8], the following limitations existed: (1) Although the clinical significance and prognosis of mixed *Candida*/bacterial BSIs versus monomicrobial candidemia were investigated, few reports focused on specific *Candida* species, such as *C. albicans*. (2) A recent study reported that patients with mixed *Candida*/bacterial BSIs had a worse prognosis than patients with monomicrobial candidemia (45.0% crude mortality vs. 17.8% crude mortality, *p* < 0.05) [8], while other studies did not observe a similar mortality rate [4–6]. The discrepancies in this clinical outcome among different studies are not understood. (3) Some risk factors associated with mixed *Candida*/bacterial BSIs, such as prolonged length of prior hospital stay (≥7 weeks), septic shock at the time of candidemia, a high acute physiology chronic health evaluation (APACHE) II score, and use of antibiotics, are supported by data mainly from Korea and Spain. Whether these risk factors also apply in other countries, such as China, is unknown. Therefore, it is necessary to investigate the clinical features of mixed *Candida*/bacterial BSIs involving specific *Candida* species in China.

Although an increase in the proportion of non-*albicans Candida* species was observed in some epidemiologic studies [9, 10], *C. albicans* is still the most common species isolated from patients with candidemia, followed by *C. glabrata* and *C. parapsilosis* [2–4, 10, 11]. The following differences were found among different *Candida* spp. (1) Different distributions were observed among different *Candida* spp. *C. albicans* is the predominant species associated with ICU-related infections, while *C. glabrata* is most commonly associated with gastrointestinal tract diseases [11]. (2) Different resistance rates to common antifungal agents have been observed between *C. albicans* and non-*albicans Candida* species. Non-*albicans Candida* species are more likely to be resistant to fluconazole than *C. albicans* [11]. (3) Different outcomes, such as mortality, also exist between non-*albicans* candidemia and *C. albicans* infection [9, 10]. Whether there are differences in the sensitivity to antifungal agents, the severity of illness or mortality between those with mixed- CA/B-BSIs and mono-CA-BSI, and which factors are associated with mixed-CA/B-BSIs are not well known. Given that *C. albicans* is the most common species responsible for candidemia and few reports about mixed-candidemia involving a specific *Candida* species, such as *C. albicans*, exist, we performed the study to investigate the clinical characteristics of, risk factors for and outcomes of mixed-CA/B-BSIs compared with mono-CA-BSI.

## Material and methods

### Patients and study design

From January 2013 to December 2018, we conducted a single-center retrospective cohort study at the Second Affiliated Hospital of Zhejiang University School of Medicine, a teaching hospital with 3200 beds in Hangzhou, China. The study was approved by the Human Ethics Board of the Ethics Committee of the Second Affiliated Hospital of Zhejiang University Medical College. Due to its retrospective nature, the Ethics Committee determined that patient consent was not required.

**Fig. 1:**
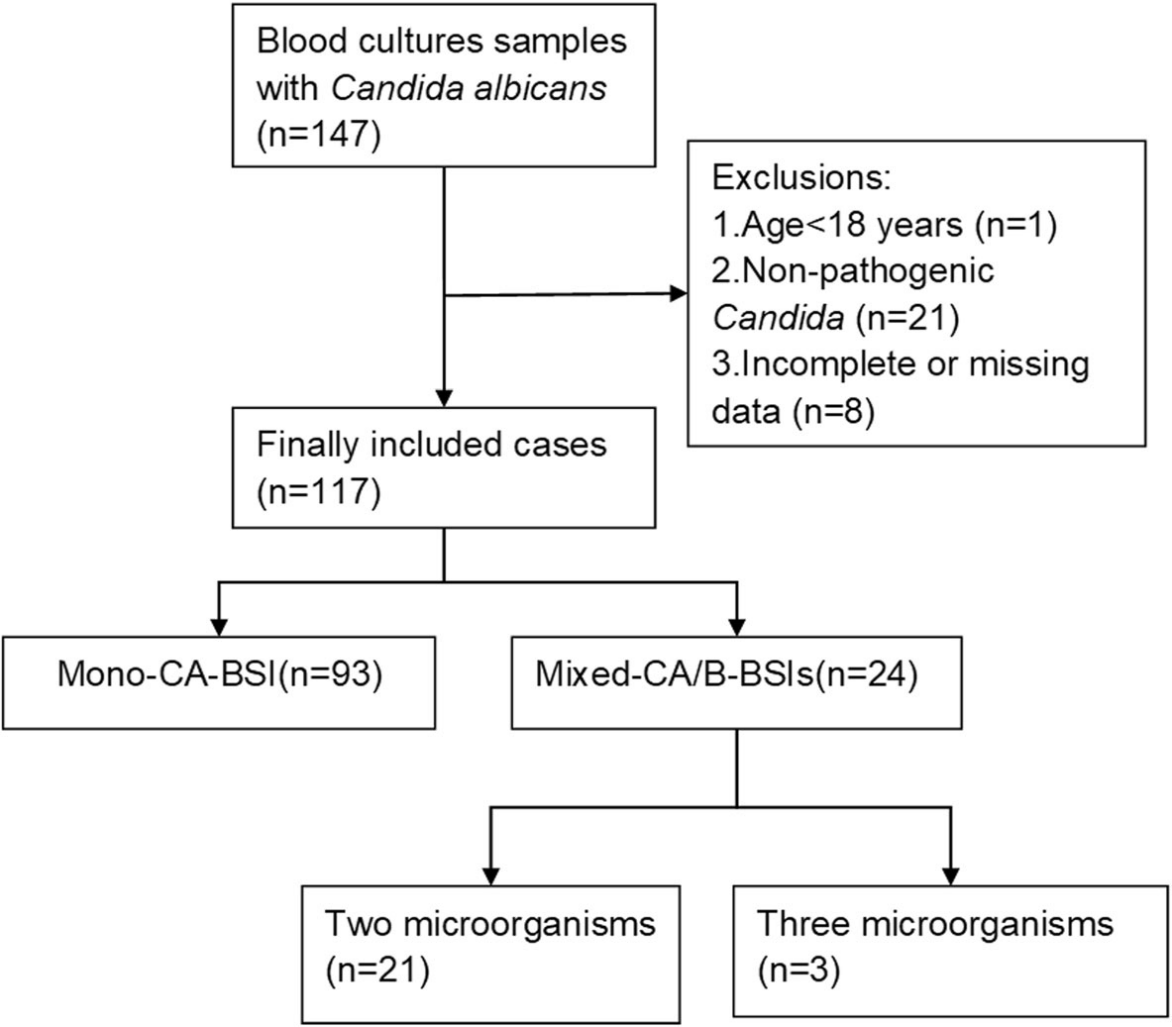
Flowchart of study participant enrollment

Patients positive for *C. albicans* according to blood culture were recruited. Candidemia that occurred 30 days after the initial episode was considered a new case [10]. The exclusion criteria were as follows: a) age < 18 years old; b) incomplete or missing case data; and c) the presence of nonpathogenic *C. albicans*. Common skin flora (e.g., *Bacillus* spp., *Corynebacterium* spp., *Micrococcus* spp., *Streptococci*, *Lactobacillus* spp. and *CNS*) were considered pathogenic only when they were present in two or more consecutive blood cultures from at least two separate blood draws or from two separate sites on the same or two consecutive calendar days. Moreover, at the time of specimen collection, the patients must have at least one of the following signs or symptoms: fever (> 38.0 °C), chills, or hypotension [12, 13]. Thus, a total of 147 blood culture specimens containing *C. albicans* were initially screened, and a final total of 117 cases were recruited, with 24 cases of mixed-CA/B-BSIs and 93 cases of mono-CA-BSI (Fig. 1).

### Data collection

Patient data were collected from electronic medical records. Patient characteristics included age, sex, severity of illness, sequential organ failure assessment (SOFA) score, and APACHE II score in the first 24 h following candidemia onset. Data regarding prior ICU stay, prior hospital stay, underlying diseases, immune status, hospitalization ward, life-sustaining treatments ≥24 h, prior use of antibiotics or antifungal agents, previous treatments such as surgical procedures, source control methods and outcomes (length of hospital stay, length of ICU stay, septic shock after the onset of BSI and 28-day mortality after the onset of BSI) were collected. Microbiological data, such as copathogens in mixed-CA/B-BSIs, likely sources of BSI and sensitivity to antimycotics, were also recorded.

### Species identification and antimycotic sensitivity test

Blood samples were collected following rigorous skin disinfection to obtain at least two sets of aerobic and anaerobic blood cultures (10–20 mL per bottle) when the patients were suspected of BSI with clinical manifestations, i.e., fever > 38.0 °C, chills, hypotension, low-grade fever at 38 °C or even hypothermia if sepsis was suspected [12]. Species identification of both bacteria and yeasts was performed by matrix-assisted laser desorption/ionization time of flight mass spectrometry (MALDI-TOF MS) (Bruker Daltonik GmbH, Bremen, Germany). Antimicrobial susceptibility testing for bacteria and yeasts was carried out with a Vitek 2 Compact system and ATB FUNGUS 3 panel (bioMerieux, France), respectively. The results for bacteria and *C. albicans* were interpreted according to breakpoints defined by the Clinical Laboratory Standards Institute [14, 15]. Because echinocandins were not included in the ATB FUNGUS 3 panel, the results of caspofungin susceptibility were unknown.

### Definitions

Candidemia was defined as the isolation of *Candida* in blood culture accompanied by fever, chills or hypotension and other corresponding clinical symptoms and signs and the exclusion of specimen contamination [16]. If the identified *Candida* species was *C. albicans*, CA-BSI was considered. Mixed-CA/B-BSIs were defined as the isolation of a bacterial organism from blood cultures obtained within 48 h before or after the onset of CA-BSI [9]. An infection was considered a healthcare-associated infection (HAI) if the date of the event (specific infection criteria) occurred on or after the 3rd calendar day of admission in an inpatient department; the day of admission to the inpatient department was regarded as calendar day 1 [13]. A definitive diagnosis of catheter-related bloodstream infection (CRBSI) required that the same organism was cultivated from at least one percutaneous blood culture and catheter tip culture or that two cultured blood samples (one from a catheter hub and the other from a peripheral vein) met the CRBSI criteria for quantitative blood culture or differential time to positivity [17]. If no other primary source of infection for candidemia can be assigned as secondary is found, a primary BSI with *Candida* is identified [12]. The timing of administration of antifungal therapy was defined as the interval between the time at which the first *C. albicans*-positive blood sample for culture was drawn and the time at which antifungal treatment was first administered [18]. Antifungal therapy was considered appropriate if the isolated *Candida* spp. was sensitive to the chosen antifungal agent and the antifungal agent was administered with an adequate dosage (for example, fluconazole was administered with a loading dose of 800 mg [12 mg/kg] followed by 400 mg [6 mg/kg] daily, and caspofungin was administered with a loading dose of 70 mg followed by 50 mg daily) [3, 19]. A delay in empiric antifungal treatment was considered when initial administration occurred more than 12 h after the first positive blood sample [18]. Appropriate antibiotic therapy was defined as antibiotic therapy for bacteremia, where applicable, and sensitivity of the pathogen to the agent [20]. Septic shock was consistent with the Third International Consensus Definitions for Sepsis and Septic Shock (Sepsis-3) [21].

### Statistical analyses

Statistical analysis was performed with SPSS 20.0 software (IBM Corp, Armonk, NY, USA). Continuous variables are presented as the means ± standard deviations if the data were normally distributed and as medians and interquartile ranges (IQRs) if the data were nonnormally distributed. Continuous variables were compared by Student’s t-test or the Mann-Whitney U test, and enumerated variables were compared by the Pearson χ^2^ or Fisher’s exact test, where appropriate. Variables with *p*-values < 0.05 in the univariate analysis were entered into the multivariable model. Continuous variables were treated as dichotomous variables based on the Youden index. The multivariate analysis was performed with logistic regression to identify independent risk factors for mixed-CA/B-BSIs. Kaplan-Meier survival estimates were used to generate the survival curves. Differences between survival curves were assessed with log-rank tests. A two-tailed *p* < 0.05 was considered statistically significant.

## Results

### Demographics and clinical characteristics

The median age was 68 years (IQR, 59–75 years), and 58.1% (68/117) of the patients were male. Mono-CA-BSI and mixed-CA/B-BSIs were responsible for 93/117 (79.5%) and 24/117 (20.5%) cases, respectively. The most common ward associated with CA-BSI occurrence was the ICU (66.7%), followed by the surgical ward (23.9%) and medical ward (9.4%). Solid tumors were the most common comorbidity (28.2%), followed by diabetes mellitus (23.9%). There were no significant differences in age, sex, immune status, or illness severity between the two groups. In the surgical patients and ICU patients, 65.8% (77/117) and 66.7% (78/117) episodes were documented, respectively. Other common predisposing factors for candidemia included CVC insertion (106/117, 90.6%), urethral catheter insertion (106/117, 90.6%), prior antibiotic exposure (93/117, 79.5%) and total parenteral nutrition (TPN) (85/117, 72.6%). Compared with the mono-CA-BSI group, the mixed-CA/B-BSIs group had a longer ICU stay before candidemia onset [12.0 (8.0,17.8) vs. 1.0 (0.0,11.0) days, *p* = 0.001], longer hospital stay before candidemia onset [19.0 (12.0,30.8) vs. 12.0 (2.0,26.5) days, *p* = 0.031], longer duration of mechanical ventilation before candidemia onset [11.0 (0.3,24.5) vs. 1.0 (0.0,10.0) days, *p* = 0.013], and longer prior antibiotic exposure before candidemia onset [17.0 (10.3,28.8) vs. 8.0 (1.0,20.5) days, *p* = 0.007]; additionally, they were more likely to have an indwelling hemodialysis catheter [41.7% vs. 18.3%, *p* = 0.015] and presence of two or more central venous catheters [50.0% vs. 25.8%, *p* = 0.022], and they had higher rates of life-sustaining treatments such as invasive mechanical ventilation (81.8% vs. 54.7%, *p* = 0.020) and continuous renal replacement therapy (CRRT) (41.7% vs. 21.5%, *p* = 0.044). Nonetheless, there were no significant differences in the proportions of surgical patients, blood transfusion, TPN, or hypoproteinemia (see Table 1). The main source of CA-BSI was CVCs (29.1%, 34/117), followed by intra-abdominal catheters (20.5%, 24/117). The main sources of mixed-CA/B-BSIs were CVCs (29.2%, 7/24) and primary sources (29.2%, 7/24). The main source of mono-CA-BSI was CVCs (29.0%, 27/93), followed by intra-abdominal catheters (19.4%, 18/93). Compared with the sources of *Candida* in mono-CA-BSI, the sources of *Candida* in mixed-CA/B-BSIs were not significantly different, as shown in Table 2. Regarding infection source control, the rate of CVC removal within 48 h after the first positive sample in the mixed CA/B-BSI group was higher than that in the mono-CA-BSI group (54.2% vs. 29.0%, *p* = 0.021), but there was no significant difference in the rate of fungal collection from drainage fluid between the two groups (20.8% vs. 15.1%, *p* = 0.708) (see Table 2).

**Table 1:** Baseline characteristics of the patients with mono-CA-BSI or mixed-CA/B-BSIs

Characteristics	Total(*n* = 117)	Mono-CA-BSI(*n* = 93)	Mixed-CA/B-BSIs(*n* = 24)	*P*value
Age, median years (IQR)	68 (59,75)	69 (59,76)	64 (47,74)	0.399
Male sex [n (%)]	68 (58.1%)	53 (56.9%)	15 (62.5%)	0.626
APACHE II score at the onset of candidemia (IQR)	17.0 (11.5,24.5)	17.0 (12.0,24.0)	17.5 (10.0,26.5)	0.863
SOFA score at the onset of candidemia (IQR)	6.0 (2.0,9.0)	5.0 (2.0,9.0)	6.5 (2.0,9.8)	0.494
Prior ICU stay (days) (IQR)	3.0 (0.0,14.0)	1.0 (0.0,11.0)	12.0 (8.0,17.8)	**0.001**
Prior hospital stay (days) (IQR)	14.0 (4.5,27.5)	12.0 (2.0,26.5)	19.0 (12.0,30.8)	**0.031**
Prior ventilation mechanical ventilation (days) (IQR)	1.0 (0.0,13.0)	1.0 (0.0,10.0)	11.0 (0.3,24.5)	**0.013**
Underlying disease [n (%)]				
Diabetes mellitus	28 (23.9%)	23 (24.7%)	5 (20.8%)	0.690
Chronic cardiac dysfunction	24 (20.5%)	16 (17.2%)	8 (33.3%)	0.144
Chronic obstructive pulmonary disease	5 (4.3%)	5 (5.4%)	0 (0%)	0.552
Chronic renal insufficiency	9 (7.7%)	8 (8.6%)	1 (4.2%)	0.766
Chronic hepatic insufficiency	14 (12.0%)	13 (14.0%)	1 (4.2%)	0.333
Solid tumour	33 (28.2%)	28 (30.1%)	5 (20.8%)	0.368
Haematological malignancy	1 (0.9%)	1 (1.1%)	0 (0%)	> 0.999
Trauma	19 (16.2%)	14 (15.1%)	5 (20.8%)	0.708
Burn injury	4 (3.4%)	2 (2.2%)	2 (8.3%)	0.186
Transplant	14 (12.0%)	11 (11.8%)	3 (12.5%)	> 0.999
Immunocompromised [n (%)]				
Immunosuppressant therapy	6 (5.1%)	6 (6.5%)	0 (0.0%)	0.448
Steroid therapy	6 (5.1%)	6 (6.5%)	0 (0.0%)	0.448
Chemotherapy/radiation	7 (6.0%)	7 (7.5%)	0 (0.0%)	0.366
Neutropenia	4 (3.4%)	3 (3.2%)	1 (4.2%)	> 0.999
Blood transfusion [n (%)]	40 (34.2%)	30 (32.2%)	10 (41.7%)	0.386
Hospitalization ward [n (%)]				
Medical	11 (9.4%)	11 (11.8%)	0 (0.0%)	0.168
Surgical	28 (23.9%)	24 (25.8%)	4 (16.7%)	0.349
ICU	78 (66.7%)	58 (62.4%)	20 (83.3%)	0.052
Nosocomial infection [n (%)]	112 (95.7%)	88 (94.6%)	24 (100%)	0.552
Life-sustaining treatments ≥24 h [n (%)]				
Invasive mechanical ventilation	65 (60.2%)	47 (54.7%)	18 (81.8%)	**0.020**
Vasopressor	45 (38.5%)	34 (36.6%)	11 (45.8%)	0.405
CRRT	30 (25.6%)	20 (21.5%)	10 (41.7%)	**0.044**
Catheterisation ^a^ [n (%)]				
Central venous catheter^b^	106 (90.6%)	83 (89.2%)	23 (95.8%)	0.553
Hemodialysis catheter^c^	27 (23.1%)	17 (18.3%)	10 (41.7%)	**0.015**
PICC	13 (11.1%)	10 (10.8%)	3 (12.5%)	> 0.999
Peripheral arterial catheters	37 (31.6%)	29 (31.2%)	8 (33.3%)	0.840
Drainage tube	77 (65.8%)	59 (63.4%)	18 (75.0%)	0.287
Urethral catheter	106 (90.6%)	85 (91.4%)	21 (87.5%)	0.848
Presence of two or more central venous catheters	36 (30.8%)	24 (25.8%)	12 (50.0%)	**0.022**
Total parenteral nutrition [n (%)]	85 (72.6%)	65 (69.9%)	20 (83.3%)	0.188
Hypoproteinemia [n (%)]	49 (41.9%)	37 (39.8%)	12 (50.0%)	0.366
Surgery [n (%)]	77 (65.8%)	59 (63.4%)	18 (75.0%)	0.287
Abdominal	39 (33.3%)	32 (34.4%)	7 (29.2%)	0.627

**Notes**: Bold, indicates *P* < 0.05

**Abbreviations**: *IQR* interquartile range, *COPD* chronic obstructive pulmonary disorder, *SOFA* sequential organ failure assessment, *APACHE* acute physiology and chronic health evaluation, *ICU* intensive care unit, *CRRT* continuous renal replacement therapy, *PICC* Peripherally inserted central catheters

^a^Included patients who were required to be catheterised within 2 weeks of the first positive sample, regardless of whether or not the catheter was removed before diagnosis

^b^Non-tunneled central venous catheters such as subclavian, internal jugular and femoral venous catheters excluding hemodialysis catheter and PICC

^c^Non-tunneled temporary dialysis catheter

**Table 2:** The source of candidemia, prior antibiotic and antifungal therapy of the mono-CA-BSI compared with the Mixed-CA/B-BSIs

Variable	Total(*n =* 117)	Mono-CA-BSI(*n =* 93)	Mixed-CA/B-BSIs(*n =* 24)	*P* value
Source of candidaemia [n (%)]				
Definitive CVC-related	34 (29.1%)	27 (29.0%)	7 (29.2%)	0.990
Intra-abdominal	24 (20.5%)	18 (19.4%)	6 (25.0%)	0.744
Primary	22 (18.8%)	15 (16.1%)	7 (29.2%)	0.244
Lower respiratory tract	12 (10.3%)	11 (11.8%)	1 (4.2%)	0.468
Urinary tract infection	7 (6.0%)	6 (6.5%)	1 (4.2%)	> 0.999
Gastrointestinal tract	6 (5.1%)	6 (6.5%)	0 (0.0%)	0.344
Skin and Soft tissue	5 (4.3%)	4 (4.3%)	1 (4.2%)	> 0.999
Meningitis	3 (2.6%)	2 (2.2%)	1 (4.2%)	0.501
Endocardium	2 (1.7%)	2 (2.2%)	0 (0.0%)	> 0.999
Osteoarthritis	1 (0.9%)	1 (1.1%)	0 (0.0%)	> 0.999
Source control [n (%)]				
Removal of contaminated lines ^a^	40 (34.2%)	27 (29.0%)	13 (54.2%)	**0.021**
Draining of fungal collection	19 (16.2%)	14 (15.1%)	5 (20.8%)	0.708
Days of prior antibiotic exposure (IQR)	11.0 (3.0,22.0)	8.0 (1.0,20.5)	17.0 (10.3,28.8)	**0.007**
Prior antibiotic exposure ^b^ [n (%)]	93 (79.5%)	69 (74.2%)	24 (100.0%)	**0.012**
Cephalosporins	33 (28.2%)	25 (26.9%)	8 (33.3%)	0.531
Carbapenems	49 (41.9%)	41 (44.1%)	8 (33.3%)	0.341
Penicillins	25 (21.4%)	19 (20.4%)	6 (25.0%)	0.626
Quinolones	4 (3.4%)	4 (4.3%)	0 (0.0%)	0.580
Initial antifungal agent [n (%)]				
Fluconazole	40 (34.2%)	32 (34.4%)	8 (33.3%)	0.921
Echinocandin	46 (39.3%)	36 (38.7%)	10 (41.7%)	0.791
Voriconazole	11 (9.4%)	9 (9.7%)	2 (8.3%)	> 0.999
Prior antifungal exposure [n (%)]	10 (8.5%)	6 (6.4%)	4 (16.7%)	0.235
Appropriate Antifungal therapy ^c^ [n (%)]	43 (36.8%)	35 (37.6%)	8 (33.3%)	0.697
Delay in initiation of empiric antifungal treatment ^d^ [n (%)]	100 (85.5%)	82 (88.2%)	18 (75.0%)	0.103

Abbreviations: *CVC* central venous catheter, *PICC* Peripherally inserted central catheters, *CRBSI* catheter-related bloodstream infection;

^a^Central venous catheter removed within 48 h after the first positive sample

^b^All patients receiving systemic drug therapy for ≥3 days within 2 weeks prior to candidaemia onset

^c^Antifungal therapy was defined as appropriate if the isolated Candida spp. was sensitive to the chosen antifungal agent, and the antifungal agent was used with adequate dosages (like Fluconazole: loading dose of 800 mg [12 mg/kg], then 400 mg [6 mg/kg] daily; Caspofungin: loading dose of 70 mg, then 50 mg daily)

^d^The delay of empiric antifungal treatment was considered as initial use more than 12 h after the report of first positive blood sample

A high rate of delay of initiation of empiric antifungal treatment (85.5%) was observed among all patients, and no difference was found between the mixed-CA/B-BSIs (75.0%) and mono-CA-BSI (88.2%) groups. In addition, the total rate of appropriate antifungal therapy was less than 50% (36.8%), and it was similar between patients with mixed-CA/B-BSIs (33.3%) and those with mono-CA-BSI (37.6%), as shown in Table 2. The proportions of empiric treatment and appropriate antibiotic therapy for bacteremia in mixed-CA/B-BSIs patients were 33% (8/24) and 70% (17/24), respectively.

### Antifungal susceptibility

The isolated *C. albicans* in both groups exhibited 100% susceptibility to amphotericin B, voriconazole, and no resistance to fluconazole was observed. Notably, in the mono-CA-BSI and mixed CA/B-BSIs groups, 11 (24.4%) and 2 (13.3%) cases were completely resistant to ketoconazole, respectively. There was no significant difference between the two groups in the in-vitro antifungal susceptibility test results, as shown in Table 3. Because the drug sensitivity kit used in our current microbiology laboratory does not include echinocandins, the specific drug sensitivity of *C. albicans* to echinocandins was unclear.

**Table 3:** In vitro antifungal susceptibility of *C. albicans* between mono-CA-BSI and mixed-CA/B-BSIs

*Candida* species	Antifungalagent	mono-CA-BSI(*n =* 93)				mixed-CA/B-BSIs(*n =* 24)				*P* value
		Number of strains	Drug sensitivity			Number of strains	Drug sensitivity			
			S	I	R		S	I	R	
*C.albicans*	Fluconazole(*n* = 104) ^a^	85(91.3%)	81(95.3%)	4(4.7%)	0	19(79.1%)	19(100.0%)	0	0	> 0.999
	Clotrimazole(*n* = 69) ^a^	55(59.1%)	54(98.2%)	0	1(1.8%)	15(62.5%)	14(93.3%)	0	0	0.901
	Ketoconazole(*n* = 60) ^a^	45(48.3%)	19(42.2%)	15(33.3%)	11(24.4%)	15(62.5%)	7(46.7%)	6(40.0%)	2(13.3%)	0.764
	Itraconazole(*n* = 111) ^a^	89(95.7%)	86(96.6%)	1(1.1%)	2(2.2%)	22(91.7%)	21(95.5%)	1(4.5%)	0	> 0.999
	Amphotericin B (*n =* 111) ^a^	90(96.8%)	90(100.0%)	0	0	21(87.5%)	21(100.0%)	0	0	> 0.999
	Nystatin(*n* = 68) ^a^	56(60.2%)	55(98.2%)	1(1.8%)	0	12(50.0%)	12(100.0%)	0	0	> 0.999
	5-fluorocytosine(*n* = 38) ^a^	31(33.3%)	30(96.8%)	0	1(3.2%)	7(29.1%)	7(100.0%)	0	0	> 0.999
	Voriconazole(*n* = 103) ^a^	82(88.2%)	82(100.0%)	0	0	21(87.5%)	21(100.0%)	0	0	> 0.999

*S* sensitive, *I* intermediary, *R* resistant

^a^Not all agents listed tested in all isolates

### Independent risk factors for mixed-CA/B-BSIs

Variables with *p*-value of < 0.05, including a prior hospital stay> 7 days, a prior ICU stay> 2 days, prior antibiotic exposure> 7 days, prior mechanical ventilation> 2 days, an indwelled hemodialysis catheter and the presence of two or more CVCs at the time of onset of candidemia, were entered into the multivariable logistic regression model to identify factors associated with mixed-CA/B-BSIs. As shown in Table 4, the only independent risk factor for mixed-CA/B-BSIs was a prior ICU stay > 2 days (adjusted odds ratio [OR], 7.445; 95% confidence interval [CI], 1.152–48.132).

**Table 4:** Multivariable logistic regression of factors associated with mixed-CA/B-BSIs

Risk factors	B	S.E.	Wald	*P* value	OR(95% CI)
Prior hospital stay> 7 days	0.787	1.581	0.248	0.618	2.198 (0.099,48.740)
Prior ICU stay> 2 days	2.008	0.952	4.444	**0.035**	7.445 (1.152,48.132)
Prior antibiotic exposure> 7 days	1.289	1.176	1.203	0.273	3.631 (0.362,36.383)
Prior mechanical ventilation> 2 days	−0.469	0.809	0.336	0.562	0.626 (0.128,3.057)
Hemodialysis catheter	0.707	0.913	0.600	0.439	2.028 (0.339,12.133)
Two or more central venous catheters	0.525	0.889	0.348	0.555	1.690 (0.296,9.652)
Constant	−4.519	1.147	15.517	0.000	0.011(−)

**Notes**: Bold, indicates *P* < 0.05

**Abbreviations**: *B* coefficient, *S.E.* standard error, *Wald* Wald test statistic, *OR* odds ratio, *CI* confidence interval, *ICU* intensive care unit, *CRRT* continuous renal replacement therapy

### Species distributions of concomitant bacteria isolated from the mixed-CA/B-BSIs

**Fig. 2:**
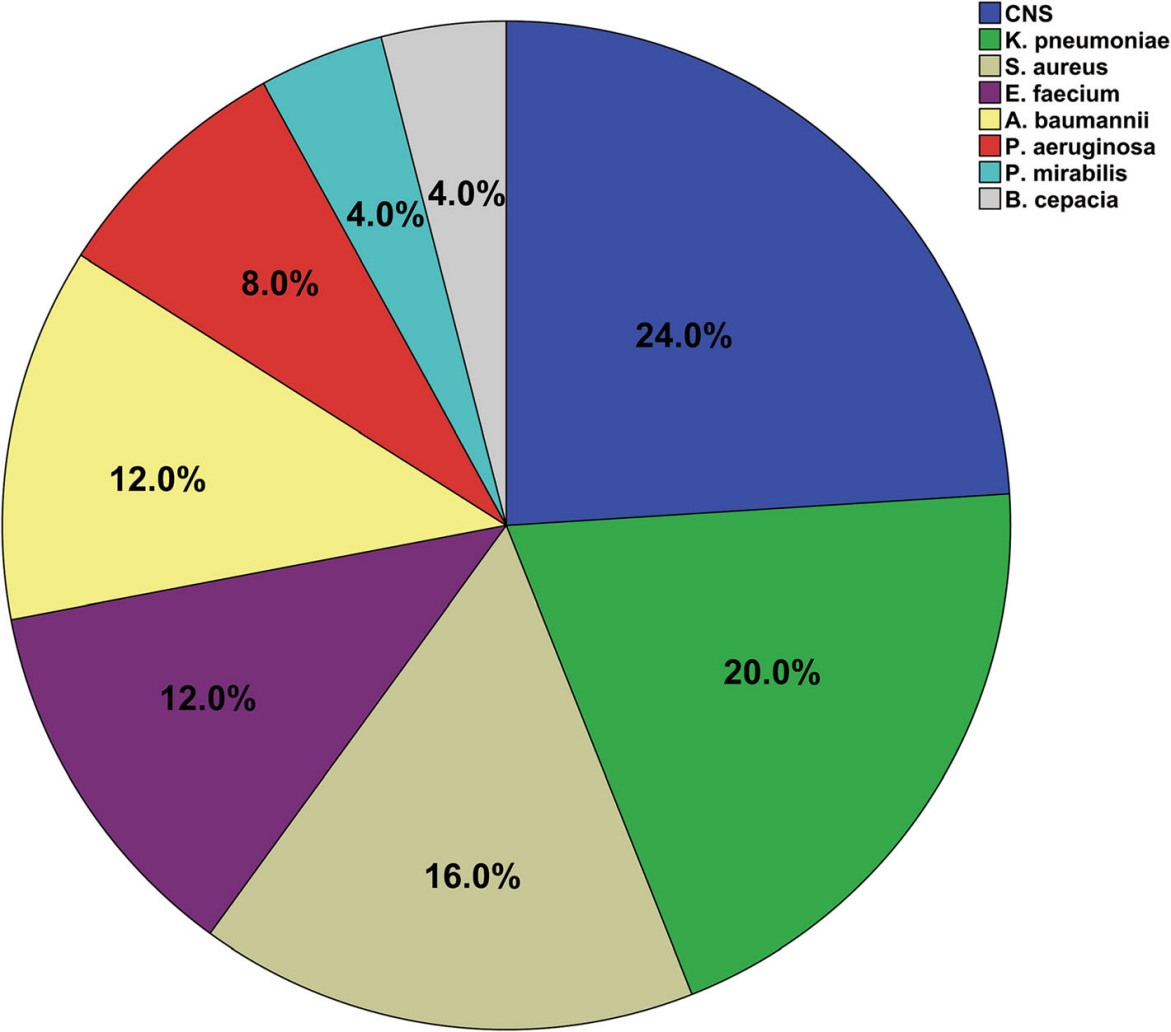
Species distributions of concomitant bacteria isolated from the mixed-CA/B-BSIs

The most common copathogens were gram-positive bacteria (52.0%), followed by gram-negative bacteria (48.0%). In terms of the exact microorganisms, the most frequent pathogen was *CNS* (24.0%), followed by *Klebsiella pneumoniae* (*K. pneumoniae*) (20.0%) and *Staphylococcus aureus* (*S. aureus*) (16.0%). The detailed distribution of concomitant bacterial species in mixed-CA/B-BSIs is shown in Fig. 2.

### Outcomes

**Fig. 3:**
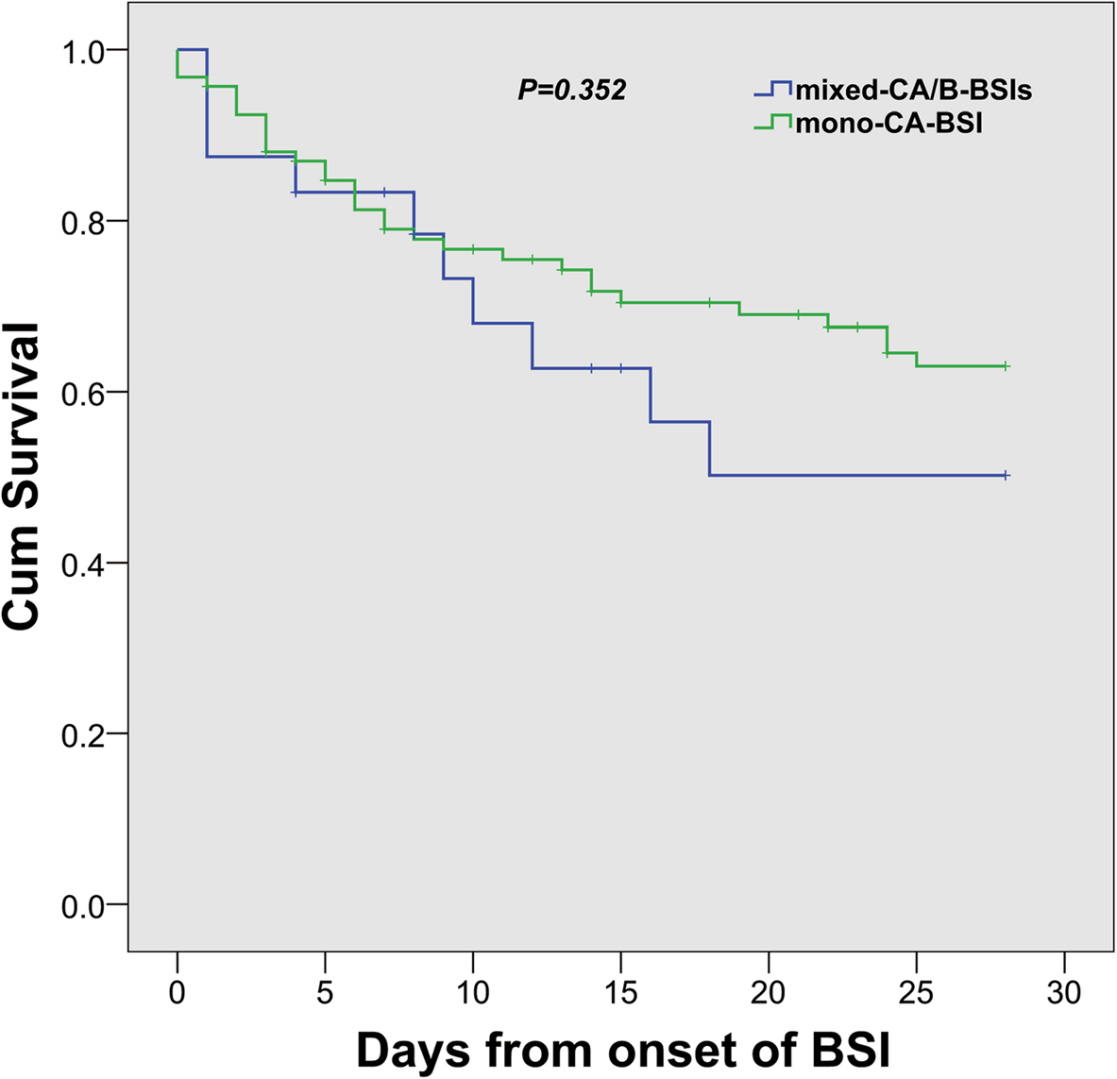
Kaplan-Meier estimates of survival in patients with mixed Candida albicans/bacterial bloodstream infections and monomicrobial Candida albicans bloodstream infection

The median length of ICU stay was 14 days (IQR, 1.0–33.0), and the median length of hospital stay was 33 days (IQR, 18.0–56.0). Compared with patients with mono-CA-BSI, patients with mixed -CA/B-BSIs had a prolonged length of ICU stay [8.0 (0.0, 31.5) vs. 22.0 (14.3, 42.2) days, *p* = 0.010] and longer mechanical ventilation time [3.0 (0.0, 24.5) vs. 17.5 (4.5, 34.8) days, *p* = 0.019]. The incidence of septic shock, 28-day and 60-day mortality, and in-hospital mortality in patients with mixed-CA/B-BSIs were not different from those in patients with mono-CA-BSI (Table 5, Fig. 3).

**Table 5:** Comparison of outcomes between mono-CA-BSI and mixed-CA/B-BSIs

Outcomes	Total(*n =* 117)	mono-CA-BSI(*n =* 93)	mixed-CA/B-BSIs(*n =* 24)	*P* value
Total ICU stay days (IQR)	14.0 (1.0, 33.0)	8.0 (0.0, 31.5)	22.0 (14.3, 42.2)	**0.010**
Total Hospitalization days (IQR)	33.0 (18.0, 56.0)	33.0 (15.0,51.0)	31.5 (23.0,66.0)	0.217
Total mechanical ventilation days (IQR)	6.0 (0.0,30.5)	3.0 (0.0,24.5)	17.5 (4.5,34.8)	**0.019**
Septic shock (n,%)	40 (34.2%)	31 (33.3%)	9 (37.5%)	0.701
28-day mortality (n,%)	41 (35.0%)	31 (33.3%)	10 (41.7%)	0.446
60-day mortality (n,%)	46 (39.3%)	34 (36.6%)	12 (50.0%)	0.229
In-hospital mortality (n,%)	50 (42.7%)	37 (39.8%)	13 (54.2%)	0.204

**Notes**: Bold, indicates *P* < 0.05

**Abbreviations**: *ICU* intensive care unit, *IQR* interquartile range

## Discussion

Polymicrobial bacteremia has been reported in previous studies, which was observed in 23.5 and 48.0% of patients with *Acinetobacter baumannii* bacteremia and *K. pneumoniae* bacteremia, respectively [22, 23]. In terms of enterococcal BSIs, 34.8% of cases (157/451) had coinfection with other pathogens, such as *CNS*, *A. baumannii*, or *K. pneumoniae* [24]. The current report found that the incidence of mixed- CA/B-BSIs was 20.5%. A similar proportion of mixed-CA/B-BSIs among CA-BSIs was reported in developed regions of Europe, such as Spain (18%) [4], Asia, such as South Korea (23%) [5], and China (20%) [8]. These results suggest that relatively high proportions of specific polymicrobial BSIs are observed not only in bacterial BSIs but also in candidemia and CA-BSIs.

Similar risk factors were found to be associated with mixed-CA/B-BSIs in previous studies [4, 5], including a prolonged ICU stay, a prolonged hospital stay before candidemia onset, antimicrobial administration, the presence of an indwelling hemodialysis catheter, the presence of two or more central venous catheters, and the existence of organ dysfunction/failure (e.g., the need for invasive mechanical ventilation or CRRT) (Table 1). However, there were no differences in the APACHE II score and SOFA score between groups (Table 1), which might reflect similar severities of comorbid diseases. Although septic shock at the time of candidemia was positively associated with mixed *Candida*/bacterial BSIs in a previous study [5], it was not independently associated with mixed *Candida*/bacterial BSIs in the current study. This might be partly explained by the similar rate of appropriate antifungal therapy in both groups and a high rate of antibiotic administration (70%) and high rate of CVC removal within 48 h after the first positive sample (54.2%) in the mixed-CA/B-BSIs group (Table 2). Previous work demonstrated that more than 20.2% of nosocomial BSIs in the ICU were polymicrobial BSIs [25, 26], which is consistent with our finding that a prior ICU stay > 2 days was an independent risk factor for mixed-CA/B-BSIs. The high incidence of polymicrobial BSI in the ICU might be explained by a suboptimal host defense altered by underlying diseases, an increased number of artificial/invasive procedures, or the application of immunosuppressive therapy in critically ill patients [26]. These results indicate that patients in the ICU are not only susceptible to BSI but also vulnerable to polymicrobial BSI, including mixed-CA/B-BSIs.

In the current study, gram-positive bacteria (52%) were the main copathogens in mixed-CA/B-BSIs, followed by gram-negative bacteria (48%). Among all the specific coexisting species, *CNS* was the predominant (24%) species, consistent with previous reports [5]. Following *CNS,* the most prevalent copathogen species were *K. pneumoniae* (20%) and *S. aureus (16%)* (Fig. 2). This might be partly explained by the fact that the primary source of mixed-CA/B-BSIs is a CVC (29.2%). It has been demonstrated that the polymicrobial biofilms formed by *C. albicans* and *Staphylococcus epidermidis* in vitro are commonly found in catheter-associated infection cases [27]. Although the main copathogen in Kim’s study was also *CNS,* the second most common pathogens were *Enterococcus* spp. and *S. aureus* [5]. The gastrointestinal tract (35%) was the most common source of mixed-CA/B-BSIs in Kim’s research, while it accounted for only 5.1% of infections in the current study (Table 2). It is well known that a common source of *enterococcal* bacteremia is the gastrointestinal tract [28]. Consistent with a previous study that found a high proportion of *K. pneumoniae* (15.2%) among BSIs [29], *K. pneumoniae* was the second most common copathogen in mixed-CA/B-BSIs in our research; this might be partly due to the fact that *K. pneumoniae* has been a leading cause of HAIs over the past few decades [30]. Consistent with a previous report [6], *S. aureus* was the third most common isolated organism in conjunction with *C. albicans* in mixed-CA/B-BSIs cases. *S. aureus* formed a substantial polymicrobial biofilm in the presence of *C. albicans* [31]*.*

Although patients with mixed-CA/B-BSIs had worse outcomes (e.g., prolonged lengths of ICU stay and prolonged mechanical ventilation time) than those with mono-CA-BSI, 28-day mortality (41.7% vs. 33.3%, *P* = 0.446), 60-day mortality (50.0% vs. 36.6%, *P =* 0.229) and in-hospital mortality (54.2% vs. 39.8%, *P =* 0.204) were similar between the two groups (Table 5, Fig. 3), similar to previous studies [4, 5, 7]. In contrast, previous studies showed polymicrobial BSI was associated with a 2.2 fold risk for increased 90-day mortality in patients with community-onset BSI [32], and also promoted an increase in 28-day mortality [33]. In our study, we found no correlation between mixed-CA/B-BSIs and mortality, which might be due to similar chronic comorbidities, a similar severity of illness at the onset of candidemia, a similar rate of fungal collection from drainage fluid, a similar delay in the initiation of empiric antifungal treatment (75% vs. 88.2% *P =* 0.697) and a similar rate of appropriate antifungal therapy administration (33.3% vs. 37.6%, *P* = 0.697) (Table 2).

The present study has some limitations. First, this was a single-center study, and therefore, the results and conclusions might be influenced by local ecology, management practices, infection control policies, and susceptibility patterns. Second, some critical factors of mixed-CA/B-BSIs might have been missed because of the retrospective design. For example, the corticosteroid dosage and treatment course were not precisely obtained; thus, the immune status due to corticosteroids was unclear. We did not get information about antifungal prophylaxis and follow-up blood cultures to confirm pathogen clearance; thus, the duration of candidemia could not be obtained accurately. Third, although culture-based methods remain the gold standard to identify the causative microorganism in sepsis cases, they are notoriously insensitive, leading to challenges in implementing early interventions [34]. Nonculture diagnostic tests, such as antigen, antibody, or β-D-glucan detection assays; polymerase chain reaction (PCR) assays; and next-generation sequencing (NGS) methods, are now being performed in clinical practice as supplements to blood culture and might provide an early and/or highly sensitive diagnosis of BSI [35–37]. Finally, although this is the first report on the risk factors for and clinical outcomes of mixed-CA/B-BSIs compared with mono-CA-BSI, the relatively small sample size may impact the confidence intervals (CIs) and analysis of risk factors. Thus, further large-scale, multicenter, prospective studies are needed.

## Conclusions

Among the total CA-BSIs, mixed-CA/B-BSIs were not rare, and *CNS* was the predominant coexisting species in mixed-CA/B-BSIs. A prior ICU stay > 2 days was an independent risk factor for mixed-CA/B-BSIs. Although there was no difference in mortality, the prognosis of adult patients with mixed-CA/B-BSIs, including prolonged length of mechanical ventilation and prolonged length of ICU stay, was worse than that in patients with mono-CA-BSI.

## Data Availability

We declare that the data supporting the conclusions of this article are fully described within the article, and the database is available from the first author (lizhong975717720@foxmail.com) upon reasonable request.

## Abbreviations

CA-BSI: *Candida albicans* bloodstream infection
mono-CA-BSI: Monomicrobial *Candida albicans* bloodstream infection
mixed-CA/B-BSIs: Mixed *Candida albicans*/bacterial bloodstream infections
BSI: Bloodstream infection
IQR: Interquartile range
CRBSI: Catheter-related bloodstream infection
CRRT: Continuous renal replacement therapy
CVC: Central venous catheter
COPD: Chronic obstructive pulmonary disorder
SOFA: Sequential organ failure assessment
APACHE: Acute physiology and chronic health evaluation
ICU: Intensive care unit
OR: Odds ratio
CI: Confidence interval
CNS: Coagulase-negative Staphylococcus
*K. pneumoniae*: *Klebsiella pneumoniae*
*S. aureus*: *Staphylococcus aureus*
A. baumannii: Acinetobacter baumannii
*E. faecium*: *Enterococcus faecium*
*P. aeruginosa*: *Pseudomonas aeruginosa*
*B. cepacia*: *Burkholderia cepacia*
*P. mirabilis*: *Proteus mirabilis*
*S. epidermidis*: *Staphylococcus epidermidis*
CLSI: Clinical and Laboratory Standards Institute
